# A new combined approach to lost medial rectus muscle retrieval using the endoscopic transnasal approach, transcutaneous medial orbitotomy, and the sub-Tenon approach

**DOI:** 10.1016/j.bjorl.2021.07.004

**Published:** 2021-10-12

**Authors:** Kosuke Takabayashi, Yohei Maeda, Hiroyuki Kagokawa, Masayoshi Nagamine, Nobuya Kataoka, Isao Ota, Taketoshi Fujita

**Affiliations:** a Japanese Red Cross Asahikawa Hospital, Department of Otorhinolaryngology, Hokkaido, Japan; b Osaka University Graduate School of Medicine, Department of Otorhinolaryngology-Head and Neck Surgery, Suita City, Osaka, Japan; c Japanese Red Cross Asahikawa Hospital, Department of Ophthalmology, Hokkaido, Japan

## Introduction

Loss of the medial rectus muscle is extremely rare. It is associated with complex pathology. Retrieval of the transected muscle, which is the preferred treatment, is quite difficult since the proximal muscle fragment is retracted posterior to the Tenon capsule. Several researchers have reported the results of retrieval and alternative procedures such as transplant, resection of the antagonist muscle, and Botulinum toxin injection for decades.^1^, ^2^, ^3^, ^4^, ^5^, ^6^ Plager and Parks reported that retrieval through the conventional sub-Tenon approach was possible in only one of ten cases, which is similar to the rate with strabismus surgery.^1^ Therefore, modified procedures such as the endoscopic transnasal approach^2^, ^7^ and the anterior orbital approach^3^, ^4^, ^5^ have been suggested, with better surgical results.

We can obtain excellent views with endoscopy and an extensive surgical field via the nasal sinuses. However, even with the endoscopic approach, there are cases where it is inevitably a blind operation with a 0-degree telescope.^2^

This is the first report that combined three techniques (endoscopic transnasal approach, transcutaneous medial orbitotomy, and sub-Tenon approach) to retrieve the lost medial rectus muscle of the orbit. We used the endoscopic transnasal approach supported by transcutaneous medial orbitotomy through a modified Lynch incision to obtain excellent visualization and improved safety during surgery.

## Case report

**Figure 1:**
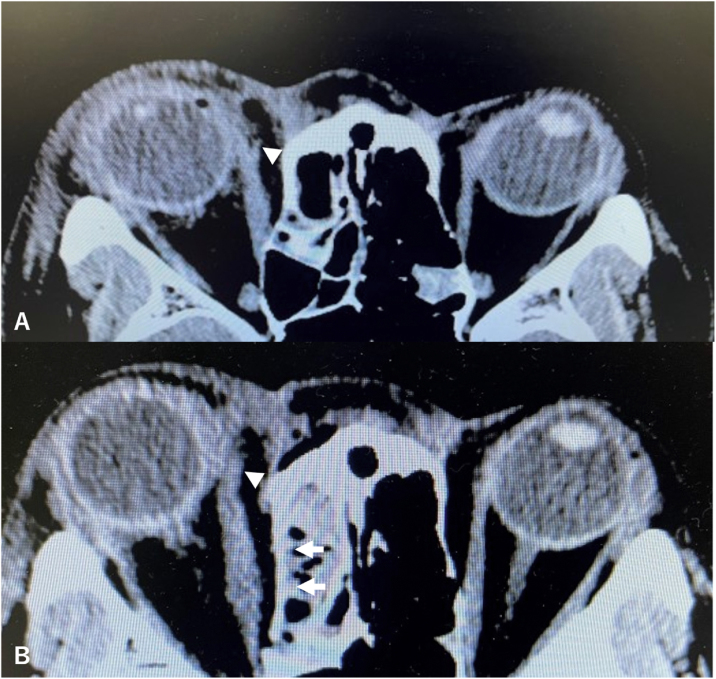
A, Preoperative axial Computed Tomography (CT) of the orbit showing transection of the medial rectus muscle. Arrowheads indicate the site of transection. B, Postoperative axial CT of the orbit showing retrieval of the medial rectus muscle. Arrowheads indicate the reconstructed medial rectus muscle. Arrows indicate the reconstructed medial wall of the orbit.

A 50-year-old woman presented emergently to the ophthalmology department with diplopia, laceration of the inner canthus, and hemorrhage in her right orbit due to a dog bite. On ophthalmological examination, she had exotropia, a laceration of the conjunctiva over the caruncle, no voluntary adduction of the globe, and transection of the superior lacrimal canaliculus in her right orbit. Due to the emergent presentation, the angle of ocular alignment was measured preoperatively with only the Hirschberg test, which showed approximately 20 degrees exotropia. There were no abnormalities in her visual acuity. Computed tomography (CT) revealed transection of the medial rectus muscle (Fig. 1A). During the first emergent surgery, the medial rectus muscle appeared transected at approximately 1 cm from the attachment to the globe, so that the proximal fragment was shifted posterior to the Tenon capsule. Ophthalmologists reconstructed the superior lacrimal canaliculus successfully, but they were not able to retrieve the medial rectus muscle using the conventional sub-Tenon approach. The main reason for unsuccessful retrieval was a narrow surgical field and blind manipulation via the conventional transorbital approach.

**Figure 2:**
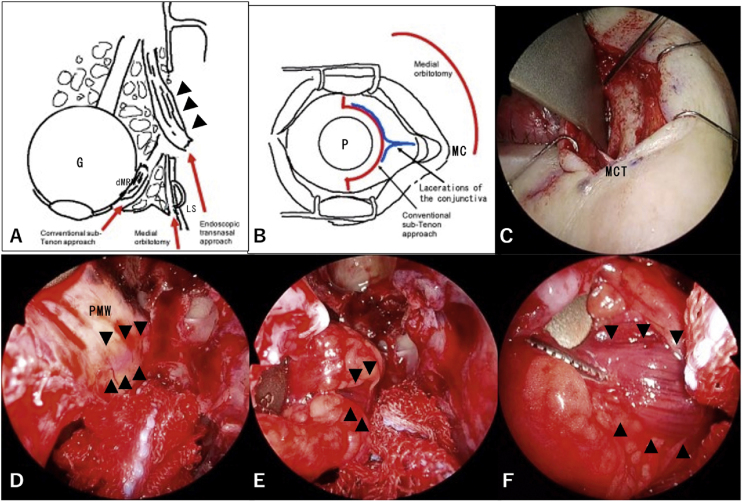
Surgical schema and images. Arrowheads show the proximal fragment of the transected medial rectus muscle. A, A combination of three different approaches is shown in the axial view. The distal fragment of the medial rectus muscle (dMRM) was recognized with the conventional sub-Tenon approach. The proximal fragment of the medial rectus muscle was recognized through the endoscopic transnasal approach supported by medial orbitotomy. B, The incision for the conventional sub-Tenon approach and the modified Lynch incision are shown as red lines. The lacerations in the conjunctiva are shown as blue lines. C, A modified Lynch incision was performed. The medial canthal tendon (MCT) was identified at the caudal end of the incision. D, The proximal fragment of the transected medial rectus muscle was identified through the periosteum of the medial wall of the orbit (PMW). View from a 0-degree telescope. E, The proximal fragment of the transected medial rectus muscle was surrounded by orbital fat. The contents of the orbit were retracted by the second surgeon with an elevator via the medial orbitotomy. View from a 0-degree telescope. F, The proximal fragment of the transected medial rectus muscle was easily recognized from the anterior view with a 70-degree angled telescope. G, Globe; LS, lacrimal Sac; MC, Medial canthus; P, Pupil.

**Figure 3:**
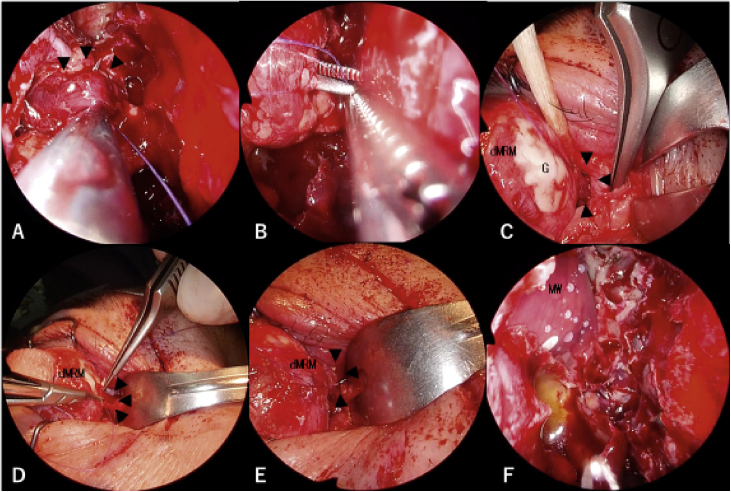
Surgical images. Arrowheads show the proximal fragment of the transected medial rectus muscle. A, A suture was hooked to the proximal fragment of the transected medial rectus muscle. View from a 0-degree telescope. B, The suture hooked to the proximal fragment of the transected medial rectus muscle was transferred to the ophthalmologist using a sub-Tenon approach. View from a 0-degree telescope. C, The proximal fragment of the transected medial rectus muscle was brought forward to the peribulbar space. (D and E) The medial rectus muscle was retrieved completely using a suture. F, The medial wall of the orbit was fixed with a silastic sheet. View from a 0-degree telescope. dMRM, Distal fragment of the medial rectus muscle; G, Globe; MW, Medial wall of the orbit.

On the next day, she was referred to our department for lost medial rectus muscle retrieval via the transnasal approach. We decided to perform reconstructive surgery urgently due to concerns about the formation of adhesions around the distal fragment of the medial rectus muscle caused by the first surgery. Three surgeons, two otorhinolaryngologists and an ophthalmologist, performed the second surgery, which was a combined procedure consisting of the endoscopic transnasal approach, transcutaneous medial orbitotomy, and conventional sub-Tenon approach (Fig. 2 A and B). Using the transnasal approach, an otorhinolaryngologist opened the frontal, ethmoid, and maxillary sinuses on the affected side to expand the surgical field and locate the medial wall of the orbit clearly. The first surgeon carefully skeletonized and removed the lamina papyracea without injuring the periorbita. At the same time, the second surgeon performed transcutaneous medial orbitotomy through a modified Lynch incision (Fig. 2C), cauterized and cut the anterior ethmoidal artery (AEA), and retracted the contents of the orbit laterally to support the first surgeon. The proximal fragment of the medial rectus muscle was recognized explicitly through the periorbita from the nasal cavity (Fig. 2D). Next, the first surgeon cut the periorbita and discovered the proximal fragment of the medial rectus muscle in the area lateral to the basal lamella (Fig. 2E and F). Of note, the medial rectus muscle was recognized clearly with the endoscope during surgery. The third surgeon used the conventional sub-Tenon approach through an incision in the ocular conjunctiva medial to the pupil and located the distal fragment of the medial rectus muscle. The third surgeon inserted forceps through the orbit into the nose on the medial layer of the medial rectus muscle with reliance on the light from the telescope inside the nose; this layer was assumed to not contain the oculomotor nerve. At the same time, the first surgeon hooked the medial rectus muscle with a suture (Fig. 3A). Next, the first surgeon transferred the suture in the nose to the third surgeon (Fig. 3B). The third surgeon brought the proximal fragment of the medial rectus muscle into the peribulbar space by pulling on the suture (Fig. 3C). The medial rectus muscle was then retrieved successfully (Fig. 3D and E). Finally, the medial wall of the orbit was patched with a silastic sheet to avoid prolapse of the orbital contents (Fig. 3F).

**Figure 4:**
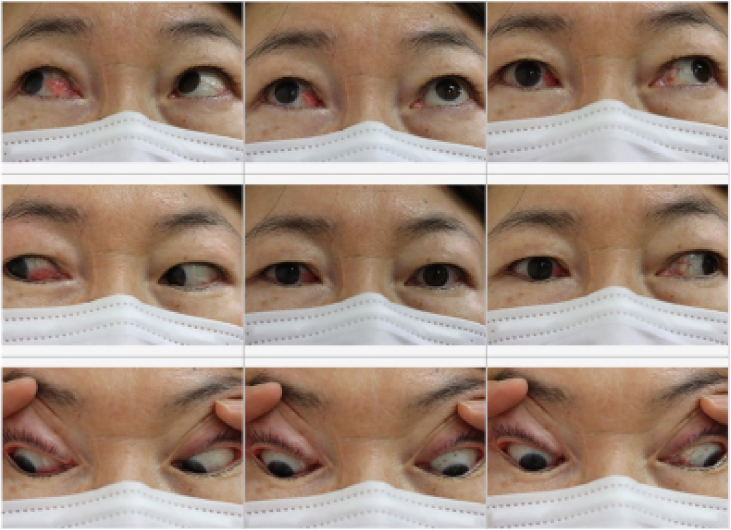
Image of the face and eye movements at 1 year after surgery. Right eye adduction improved, and no diplopia was seen during primary gaze. The scar from the modified Lynch incision was barely noticeable.

The postoperative CT scan showed successful reconstruction of the medial rectus muscle (Fig. 1B). The postoperative angle of ocular alignment at 3 months after surgery was approximately orthotropic based on the Hirschberg test and 54 Prism Diopters (PD) based on the Krimsky test. At 1 year after surgery, the angle of ocular alignment was 38 PD and no diplopia was seen during primary gaze. The scar from the modified Lynch incision was barely noticeable (Fig. 4). Therefore, she is not expected to require any additional surgeries or botulinum toxin injections for residual horizontal strabismus.

## Discussion

In this case, we selected a combination of three techniques: the endoscopic transnasal approach, transcutaneous medial orbitotomy, and the conventional sub-Tenon approach. This strategy combines the benefits of each technique. First, the endoscopic transnasal approach provides excellent views and an expanded surgical field.^2^, ^7^ In addition, a 70-degree angled telescope enabled us to maintain visualization of the medial rectus muscle during the operation because we were able to directly visualize the medial wall of the orbit. Second, a transcutaneous medial orbitotomy through a modified Lynch incision provided lateral traction of orbital contents, simplifying reconstruction of the orbital wall.^2^ Therefore, this combined approach was quite effective for safely searching for the lost medial rectus muscle and accurately reconstructing the medial wall of the orbit.

However, this approach has some drawbacks. It is necessary to assemble an interdisciplinary team of otolaryngologists and ophthalmologists, open a normal nasal cavity, and reconstruct the medial wall of the orbit. It also leaves a scar on the face. In summary, we recognize that this approach may provide great advantages despite some drawbacks.

The anterior orbital approach, a major approach for retrieval of a lost medial rectus muscle, has several advantages over the conventional sub-Tenon approach. Surgeons are able to achieve an expanded view with the transconjunctival and transcaruncular approaches and search for the lost medial rectus muscle under the periosteum without interference from orbital fat.^3^, ^4^, ^5^ In addition, the anterior orbital approach does not require reconstruction of the orbital wall, surgery on a normal nasal cavity, or an interdisciplinary team.^2^ However, due to the conical shape of the orbit, anterior orbitotomy alone may increase the risk of optic nerve injury relative to the orbital approach combined with the endoscopic transnasal approach.^8^ Therefore, our approach is safer than the anterior orbital approach. Additionally, in the current case, we were concerned about surgical manipulation through a trascaruncular approach causing damage to the conjunctiva, since lacerations of the conjunctiva by the dog bite were close to where incision would be needed for the transcaruncular approach. Hence, we selected the endoscopic transnasal approach supported by transcutaneous medial orbitotomy through a modified Lynch incision (Fig. 2B).

In general, retrieval of a medial rectus muscle is more challenging than retrieval of the other extraocular muscles because the medial rectus muscle is the only free muscle that does not have any adjacent oblique muscles. Therefore, once the medial rectus muscle is transected, it is drawn into the sub-Tenon space.^1^, ^2^, ^5^, ^6^ Even in this situation, we were able to identify the lost medial rectus muscle without any difficulty with an endoscope from the nasal cavity.

The frequency of extraocular muscle lacerations associated with orbital trauma, eyelid laceration, or strabismus surgery is highest for the medial rectus muscle.^4^, ^9^ One reason is that the attachment of the medial rectus muscle to the globe is anterior to the attachment of the other rectus muscles. Another reason is Bell’s phenomenon, which is a defensive mechanism where the eyes move upward and outward when they are closed with fear.^10^ In this case, the location of the dog bite might have been the main cause; Bell’s phenomenon might have affected the transection of the medial rectus muscle.

In the current case, we recognized the advantages of combining these three types of approaches. We have gained benefits with a combined approach. The scar from the modified Lynch incision was barely noticeable. We hope that this approach will be a useful option for surgeons who need to retrieve a transected medial rectus muscle of the orbit.

## Conclusion

We successfully retrieved a transected medial rectus muscle, which is an extremely rare and challenging case caused by a dog bite. The endoscopic transnasal approach supported by transcutaneous medial orbitotomy provided an excellent view and a safer operation.

## Funding

This report was not supported by any organization.

## Conflicts of interest

The authors declare no conflicts of interest.

## Acknowledgements

We are grateful to Dr. Meiho Nakayama for motivating us to engage in this report.

## References

[1] Plager D.A., Parks M.M. (1990). Recognition and repair of the “lost” rectus muscle. A report of 25 cases. Ophthalmology.

[2] Lenart T.D., Reichman O.S., McMahon S.J., Lambert S.R. (2000). Retrieval of lost medial rectus muscles with a combined ophthalmologic and otolaryngologic surgical approach. Am J Ophthalmol.

[3] Underdahl J.P., Demer J.L., Goldberg R.L., Rosenbaum A.L. (2001). Orbital wall approach with preoperative orbital imaging for identification and retrieval of lost or transected extraocular muscles. J AAPOS.

[4] Yen K.G., Yen M.T. (2009). Orbital approach for retrieval of transected extraocular muscles. Strabismus.

[5] Pineles S.L., Laursen J., Goldberg R.A., Demer J.L., Velez F.G. (2012). Function of transected or avulsed rectus muscles following recovery using an anterior orbitotomy approach. J AAPOS.

[6] MacEwen C.J., Lee J.P., Fells P. (1992). An etiology and management of the’ detached’ rectus muscle. Br J Ophthalmol.

[7] McKeown C.A., Metson R.B., Dunya I.M., Shore J.W., Joseph M.P. (1996). Transnasal endoscopic approach to expose the MR from the annulus of Zinn to the penetration of Tenon’s capsule. J Pediatr Ophthalmol Strabismus.

[8] Hwang S.H., Joo Y.H., Seo J.H., Kim S.W., Kang J.M. (2012). Endoscopic endonasal approach of the medial intraconal space: ct analysis of the anatomic relation between paranasal structures and orbital contents. J Craniofac Surg.

[9] Paysse E.A., Saunders R.A., Coats D.K. (2000). Surgical management of strabismus after rupture of the inferior rectus muscle. J AAPOS.

[10] Lenart T.D., Lambert S.R. (2001). Slipped and lost extraocular muscles. Ophthalmol Clin North Am.

